# 

**Published:** 1855-04

**Authors:** 


VOL. IV. NEW SERIES. No. 4.

### THE NORTH-WESTERN

From J. T. White, Casey Ill.
With Compliments

### MEDICAL AND SURGICAL JOURNAL:

EDITED BY

N. S. DAVIS, M.D.,

PROFESSOR OF PATHOLOGY, PRACTICE OF MEDICINE AND CLINICAL MEDICINE

AND

H. A. JOHNSON, A.M., M.D.

LATE LECTURER ON PHYSIOLOGY AND HISTOLOGY IN RUSH MEDICAL COLLEGE.

APRIL, 1855.

CHICAGO:

J. F. BALLANTYNE, BOOK AND JOB PRINTER,

NEW POST-OFFICE BUILDINGS, COR. OF CLARK AND RANDOLPH-STS.,

OPPOSITE THE SHERMAN HOUSE.

